# Risk Factors for Depression in Children and Adolescents with High Functioning Autism Spectrum Disorders

**DOI:** 10.1155/2015/127853

**Published:** 2015-08-25

**Authors:** Myriam De-la-Iglesia, José-Sixto Olivar

**Affiliations:** ^1^Departamento de Psicología, Facultad de Educación, Universidad de Valladolid, Campus María Zambrano, Plaza Alto de los Leones 1, 40005 Segovia, Spain; ^2^Laboratoire LPPS, EA 4057, 92100 Paris, France; ^3^Departamento de Psicología, Facultad de Educación y Trabajo Social, Universidad de Valladolid, Campus Miguel Delibes, Paseo de Belén 1, 47011 Valladolid, Spain

## Abstract

The objective of our study was to examine, discuss, and provide proposals on diagnostic comorbidity of depression in children and adolescents with high functioning autism spectrum disorder (HFASD) in the following aspects. (1) *Prevalence*. It was concluded that there are an elevated depression rate and the need for longitudinal studies to determine prevalence and incidence based on functioning level, autistic symptoms, gender, age, type of depression, prognosis, duration, and treatment. (2) *Explicative Hypotheses and Vulnerability*. The factors that present the greatest specific risk are higher cognitive functioning, self-awareness of deficit, capacity for introspection, stressful life events, adolescence, quality of social relationships, and alexithymia. (3) *Risk of Suicide*. The need for control and detection of suicidal tendencies and bullying is emphasised. (4) *Depressive Symptoms*. Indicators for early detection are proposed and their overlap with HFASD is analysed, examining the *assessment techniques* used and arguing that specific adapted tests are needed.

## 1. Introduction

The new Diagnostic and Statistical Manual of Mental Disorders, Edition 5 (DSM-5 [1]), proposes Autism Spectrum Disorder (ASD) as the only diagnosis possible for the previous category of Pervasive Developmental Disorder (DSM-IV-TR [2]), as well as the elimination of Asperger Syndrome (AS). This change in name emphasises the dimensional consideration of the clinical picture in the various areas affected (social communication and mental inflexibility) and the difficulty of establishing precise limits between the subgroups within the same category. This new classification is based on empirical evidence that the majority of individuals with a diagnosis of AS meet the DSM-IV criteria for Autistic Disorder [3, 4]. However, several research teams hold different perspectives about the new DSM-5 classification [5].

In addition, there is a consensus as to the need to go deeper in reconsidering not only diagnostic issues, but also comorbidity, assessment, and intervention in ASD [6]. Individuals with ASD show significant variability in symptom expression and in the level of cognitive functioning (DSM-5 [1]). In addition, their condition can coexist with other disorders [7]. The studies that confirm the high risk of comorbidity with depression are notable. Fundamentally, in adolescence, individuals with high functioning autism spectrum disorders (HFASD) usually present a high degree of anxiety, stress, and depression, with mood disorders being the most common comorbidity (e.g., [8]). However, because the main symptoms of ASD cause significant impairment, other psychopathological symptoms are usually not the primary focus of screening, diagnosis, or treatment. Research suggests that such conditions can exacerbate the core ASD symptoms, compromising quality of life and long-term functioning level even more [9].

That is why there is a need for specialised attention to prevent the possible appearance of depressive symptomatology, for early detection and for specific intervention if depression has appeared. However, studies that investigate the variables related to depression in autism are rare and their findings are inconsistent. This makes the work of experts in early detection and intervention more difficult. Furthermore, knowledge about the pattern of psychiatric comorbidity is scant. In spite of demonstrated idiosyncrasy in alarm signals, conclusive comprehensive studies that help to detect or lessen this risk are rare.

*Study Objectives.* In this study we carried out a critical review of scientific research on diagnostic comorbidity of HFASD and depression to ascertain the current state of the matter. We synthesised the main studies performed to date, critically analysing the findings that make it possible to improve early detection. Attempting to enhance professional praxis in prevention, early detection, and early intervention, we consequently proposed the following: (1) presenting the* rates of depression* in children and adolescents with HFASD; (2) analysing the possible* risk factors and explicative hypotheses* for diagnostic and/or symptomatological comorbidity of HFASD and depression, establishing a possible working hypothesis that supports studying the profiles of potential vulnerability and contributing factors; (3) assessing the implications of depression in HFASD (the* risk of suicide*); and (4) analysing the difficulties involved in* assessing* depression in children and adolescents with HFASD and proposing alternatives. All of these would make it possible to elaborate a proposal, in agreement with the results obtained from these analyses, that promotes and establishes possible future research paths, which would complement, refute, or confirm the studies carried out to date.

*Study Hypothesis.* This study established the hypothesis that the individuals with HFASD with higher cognitive levels and capacity for introspection would present greater risk of depression in adolescence, because they are more conscious of their difficulties with social relationships (e.g., [3, 4]). This risk would increase when the social interactions were more conflictive and these individuals could respond with suicide attempts. This hypothesis was chosen with the aims of improving prevention, as well as early detection and intervention, in depression and of attempting to clarify the most vulnerable target population.

## 2. Materials and Methods

### 2.1. Literature Search

A systematic search of computerized databases (PsyInfo, Pubmed, Web of Science, and ERIC) was undertaken using the words “Autism,” “Asperger,” “Pervasive Development Disorder,” and “PDD” in various combinations with the words “depression,” “depressive disorder,” “suicide,” “risk,” “comorbid disorder,” “vulnerability,” “comorbidity,” “prevalence,” “psychiatric disorder,” and “psychological disorder.” The abstracts were reviewed by the first author for relevance. Abstracts were considered relevant if they described their sample as having ASD and if they reported a depression measure. Next, the reference sections for data-based papers not found by the computer search were checked.

### 2.2. Selection of Studies

The studies had to meet the inclusion criteria: 2010-March 2015 studies had to report on children and adolescents (participants' age: 3–20) with diagnosed ASD, autism, or HFASD. Our paper reviews and analyses the research on the topic of interest over the last five years, including as yet unpublished studies concerning risk factors for depression in children and adolescents in the framework of the new diagnostic classification for ASD consistent with DSM-5 criteria [1].

## 3. Results

Next, we present a summary for each section of the results analysed in this study, such as summaries about rates of depression in children and adolescents with HFASD, and the factors of risk studied: (a) histories of first-degree relatives and environmental context; (b) gender; (c) age; (d) cognitive level, capacity for introspection, awareness of deficits (insight), and alexithymia; (e) social support: quality of social relationships; (f) life events and the domain of repetitive and restricted behaviours and thoughts; and (g) biological factors and comorbidity.

### 3.1. Rates of Depression in High Functioning Autism Spectrum Disorders

Depression is common in prevalence in ASD, which has increased the growing interest in understanding the nature of depression in this group (e.g., [10, 36]). In addition, these problems could influence the family negatively, producing an increase in stress and conflicts, which are high in autistic people's caregivers [37]. However, there are no large population studies that assess the incidence and prevalence of depression in ASD. Estimates on prevalence of comorbid depression vary widely among studies. In the next few years we will be able to assess the empirical effects of the new conceptual framework for ASD resulting from the DSM-5 criteria [38] in the general context of the epidemiology of depression in ASD.

Links between affective disorders and autism have been suggested for decades [39] (see Table 1). The rates are very high, both in the early research, consisting basically of case studies [11, 12], when the study objective was analysing the patterns of prevalence of disorders coexisting with HFASD [7, 13, 14, 40], independently of the age groups studied [15–18], and when the levels of depressive symptomatology of individuals with HFASD are compared with peer control groups, neurotypical (NT) individuals, [10, 19–22] or with other clinical groups (e.g., with intellectual disability [23]).

### 3.2. Risk Factors and Explicative Hypotheses for Diagnostic and/or Symptomatological Comorbidity of HFASD and Depression

In the case of individuals with HFASD, the factors that lead to them having a greater risk of coexisting psychiatric symptoms (such as depression) are still not completely understood. However, based on preliminary reports, some generalisations can be made. We present studies on risk factors and related protective factors, which will make it possible to verify or refute our working hypothesis. Specifically, based on the biopsychosocial model, factors related to depression can be seen from all spheres, which are as much psychological as well as biological and social.

*(a) Histories of First-Degree Relatives and Environmental Context.* In the general population, familial-genetic factors play an important role in the aetiology of depression in both children and adults [41]. When we are dealing with children with ASD, studies also recognise that (just as in the NT population) children with autism that have depression are more likely to have a family history of depression. For example, when Ghaziuddin's team compared the family histories of 13 participants with ASD and depression to 10 children with ASD lacking a previous recurrent history of depression, they found that 77% of the first group had a family history of depression but the percentage dropped to 30% in the group of children without depression [13].

Studies suggest greater prevalence of affective disorders in first-degree relatives with ASD [42] than in control NT individuals or those with Down's syndrome [43] and individuals with nonautistic developmental disorders [44]. This does not seem to be a reaction to having a child with ASD, given that the onset of the mood in most of the cases was registered before the individual with ASD was born [44]. Furthermore, it has been shown that parents of children with ASD and altered serotonin levels score high in depression [45], which suggests a possible interaction of shared neurochemical factors between depression and autism, at least in some cases. A recent study on 33,332 cases and 27,888 controls of European descent is interesting in this regard, as it found specific variations that underlie the genetic effects shared among ASD, schizophrenia, bipolar disorder, and major depressive disorder [26].

Many other psychosocial factors, especially those family-related (i.e., the parent-child relationship and parenting style) and their influences, may be important. The work by Rezendes and Scarpa [46] examines the roles of parenting stress and parenting self-efficacy as mediators between child behaviour problems and parental anxiety/depression, using a sample of 134 mothers with an average age of 39 and children with ASD within the age range 3–16 years. The research suggests that parental factors may influence the relationship between child behaviour problems and parenting stress and depression, including the role of parenting self-efficacy within the parenting experience, thus bringing about a two-way relationship between the parents' stress factors and the behavioural problems of children with ASD.

*(b) Gender.* When studying this variable in individuals with ASD, most authors fail to find gender differences [20, 47] or significant relationships between depressive symptoms, gender, ethnic group, or father's occupation [17]. The lack of gender effects is even more notable when we consider that they are indeed present in the general population. For this reason, the authors postulate that the absence of gender effects in autism (in contrast with the general population) “could reflect shared neurobiological deterioration,” independently of sex and “the predominant influence of the dysfunction of the organic brain in psychopathology” [23, p. 867-868]. This would imply a greater relative weight of organic factors in the appearance of depression in this collective.

*(c) Age*. A study on 34 cases with HFASD reported the presence of clinical depression in both adolescence and in adulthood, and that these characteristics seem to be related to the fact that the individuals are painfully consciousness of their handicap and of being different from other people [12, p. 118].

Current studies confirm that depression is more common in adolescents and adults than in children [23, 40]. In addition, the majority of the studies on children with autism show that the older the child and the greater the intelligence quotient (IQ), the greater the incidence of depression [23, 48]. Specifically, research on this topic [17] has found that depression is positively related to IQ, but also, especially, to age. Those authors state that this psychopathological pattern begins very early in children with autism and that, independently of the level of cognitive functioning, adolescents present greater depression than preadolescents, who in turn are more depressed than preschool age children. The authors explain these results based on the assumption that individuals of greater age and/or IQ might have greater capacity for introspection.

Studies such as the one conducted by Vickerstaff's team [48] also find that the older the youngsters with HFASD, the greater the self-reporting of symptoms of depression. However, this study adds the finding that there is a direct correlation with the fact that those who are older have greater subjective perception of difficulties in social competency. It is possible that society's expectations of these individuals begin to have a greater effect in adolescence and adulthood. The stress caused by the transition to adulthood can add even more difficulties in relating to peers. Adolescents and adults with ASD may be aware that they are behind their peers developmentally and desire to achieve milestones of development typical of adults, but they lack the adaptive skills or the executive planning abilities to take the steps needed to develop an independent life. Feelings of extreme depression may also affect the motivation required to make these life changes.

*(d) Cognitive Level, Capacity for Introspection, Awareness of Deficits (Insight), and Alexithymia.* Several studies have specifically investigated the relationship between different IQ levels, symptoms of autism, and symptoms of depression in individuals with ASD. Examples are [49] with 1,202 children with ASD aged between 4 and 17 years and with a wide range of intellectual levels [17, 30]. Based on these results it appears that a higher cognitive level and less serious symptoms within the spectrum predict a greater risk of depression.

The majority of higher functioning individuals with ASD are aware of their social difficulties and this awareness may in turn lead to greater pathology and the development of comorbid psychiatric problems [50]. Some authors [12, 39] initially suggested a possible relationship between greater self-consciousness of social problems and depression. This author indicated that young people with ASD that are sufficiently conscious of themselves and of their social difficulties tend to experience greater emotional pain when faced with social failures.

Supporting the hypothesis that higher IQ also increases social comparison and consequently deficit insight, [51, 52] gave information on the relation between the processes of social comparison and depressive symptoms in 36 adolescents with HFASD aged between 10 and 16 years. When this greater consciousness of their failures in social life is added to assumption of responsibility and internalisation of the negative events, it contributes to lowering self-esteem and increasing discouragement, which can also increase the risk for depression in individuals with ASD [15]. Meyer's team [22] found a significant correlation between depressive symptoms and social comparison scale with children aged between 7 and 13 years with HFASD when they assessed whether certain patterns of social information processing and social attribution were related to depression in the youngsters with ASD. The participants demonstrated the capacity to interpret and make attributions about social situations and to report their lower satisfaction and competence in interpersonal relationships as well as their own emotional and social difficulties. These findings represent the confirmation that many children with HFASD are aware of their social difficulties and their differences with respect to peers and that they are capable of showing enough introspection to self-report their problems.

Nevertheless, adults with ASD have alexithymia (understood as the incapability to identify, describe, and interpret emotional states) to a greater extent than the general population [53]. Alexithymia is frequently present in depression [54]. For that reason, some research teams are focusing on the differential diagnosis between HFASD and alexithymia, due to some similarities in their clinical presentations [55].

*(e) Social Support: Quality of Social Relationships.* Children and adolescents without friends and with poor quality social relationships and friendships run the risk of loneliness, stress, depression, negative affect, and concomitant developmental psychopathologies [56, 57]. For this reason, it is possible that the feeling of loneliness, fuelled by the poor social relationships that individuals with ASD have, contributes to greater levels of depression. There is evidence that social disabilities correlate with a negative state of mind in children and adolescents with HFASD [52, 58], and specifically with anxiety and depression [59] Despite this, few studies have examined the relation between these problems and the social impairment that is at the core of the disorder.

In fact, individuals with ASD and autism are traditionally conceived to have no problems with or even prefer social isolation. For example, Kanner [11, p. 249] described individuals with ASD as having a “powerful desire” for aloneness (p. 249). However, research suggests that individuals with HFASD, especially those with Asperger's Disorder, have more interest in social interaction but lack the abilities needed for success in social relationships [60]. Individuals with HFASD are integrated into their groups of reference and, consequently, are going to experience greater exposure to their peers and to social stimulation than individuals with a lower level of cognitive functioning. This stress, which involves an increase in social demands, can lead to symptoms of depression [17]. Specifically, studies on the internalisation of problems are beginning to reveal that there is a two-way relationship between negative social self-perception and difficulties with peers and that the combination of these factors predicts an increase in depression [61, 62]. For example, there is evidence that NT children rejected by their peers experience higher levels of anxiety and depression [63] and that, in individuals with ASD as well, a history of nonsuccessful interactions and social isolation can contribute to low self-esteem, frustration, and depression [50].

In NT individuals, there seems to be a protective function in children that have at least one friend. These children show higher self-esteem, less anxiety, less loneliness, and fewer episodes of victimisation [64]. In contrast, Mazurek and Kanne [49] found that, when they investigated the relation between friendship, seriousness of autistic symptoms, and depression, the high rates of depression obtained were shown in children with poor quality friendships. They also found that the absence of friendships (children with few, very poor quality or no friendships) could protect against emotional problems.

It is relevant to emphasise that there are few studies on how children with ASD perceive friendships or their lack or whether such friendships can carry out a protective function similar to that found in the reference population. As we will see, current results indicate that adolescents with HFASD generally have poorer quality friendships than their NT peer group, less intrinsic motivation to engage in friendships, and elevated levels of loneliness and depressive symptoms. These associations between friendship, lonesomeness, and depression indicate that the development of meaningful relationships may have significant effects on mental health in this population. This leads to the importance of intervention in this area for improving prevention. However, the reverse interpretation may also be true (i.e., depression may negatively affect the ability to develop relationships with others). It is also possible that there is not a causal relationship (i.e., both may be independently related to autism, which would explain the correlation between the two). Of course, all three explanations may be operating simultaneously. The need for controlled prospective studies to empirically determine risk factors should be listed as a future research necessity.

The team of Bauminger [35, 65, 66] carried out a series of studies that sought to quantify the impact of poor quality social relationships on the emotional functioning of children with HFASD. In all the series, the children with autism perceived their friendships to be of poorer quality than the NT control group did, and they reported greater feelings of loneliness. In the first of the series, Bauminger and Kasari examined the perceptions of friendship and the experiences of loneliness in a sample of children aged from 8 to 14 years with HFASD, compared to a control group paired in age and IQ [35]. The authors found high loneliness levels and friendships of poor quality. The results indicated that children with autism also differed in their understanding of friendship, which they defined in terms of company, safety, confidence, and friendliness. These children also seemed to understand loneliness in a different way (defining it from a cognitive rather than an emotional viewpoint), showing fewer associations between friendship and loneliness than the NT children. It consequently seems that children with autism may lack the capacity to link the possibility of reducing loneliness to friendship [35, 67]. A year later, the same team confirmed that the children and teens with HFASD self-reported more feelings of both social and emotional loneliness than the NT control group. This led them to suggest that young people with ASD (independently of whether or not they experience anxiety-provoking situations) may tend to feel emotionally disconnected or alone [65]. However, in a later study these authors concluded that friendship has a protective function for children with HFASD, given that greater fellowship, intimacy, and proximity with a friend are associated with higher levels of self-esteem [66].

The team of Whitehouse [10] linked the poorer quality of friendship in adolescents with HFASD to greater loneliness and greater depressive symptoms with respect to the comparison group. This finding complements work [35] showing that these feelings of loneliness indicate greater vulnerability to depression if low quality friendships are accompanied by feelings of social isolation.

*(f) Life Events and the Domain of Repetitive and Restricted Behaviours and Thoughts.* Negative life events play an important role in precipitating depression [56]. Negative events such as parents getting divorced, illnesses, death, frequent parental discord, or changing residences have been related to clinical depression, both in children and in adults [68]. Studies carried out with individuals with ASD find similar results, which suggest that children with ASD and depression experience more negative events compared to controls [69]. For example, in a study with 11 children with ASD and depression, the team for Ghaziuddin found that the depressed group had experienced an overload of adverse life events (e.g., losses significant for the child, divorce, or parental separation) in the 12 months before depression onset [70].

As for the type of negative events related to the risk of depression in this collective, we analysed the possible influence of the “age” factor in a previous section, concluding that adolescence constitutes one of the risk factors over which the greatest scientific agreement exists. We emphasise that, although this variable belongs to the biological sphere, it seems that the psychosocial area is where we can understand its possible risk effect, as a stressful life experience. This is true insofar as aging constitutes a developmental moment in which social demands increase, along with self-awareness of the difficulties involved in facing these demands.

Another stressful life event of risk for individuals with ASD is peer abuse, which studies have linked to symptoms of depression, anxiety, loneliness, and suicide [71]. Bullying frequently occurs among children with ASD. For example, 34 parents of children aged between 5 and 21 years with ASD indicated that approximately 65% had been victims of their peers [72]; in the study by the Mayes team [73] in their sample of 791 children with ASD, 57% of the mothers reported mocking and bullying. Research conducted by Wainscot and his team [74] found similar rates among 57 young people with HFASD. Other studies report rates that are even higher, reaching 75% of the adolescents with HFASD [75] or 94% when mothers of children with HFASD or with nonverbal learning disabilities were asked [76]. Studies generally indicate that between 44% and 77% of these young people have experienced bullying in the last month [32, 33, 72, 74]. This is very much higher than the rates found among NT individuals, in which they are about 10%–20% [77]. Within the spectrum of individuals with ASD, those with a higher level of functioning present greater risk than those affected with intellectual limitations. The reason appears to be that the greater severity of the symptoms accentuates the prosocial attitudes of the peers [78], but it is also because individuals affected more greatly usually function in special education centres [33].

Most authors attempt to explain these high rates by many factors related to the core symptomatology or symptoms associated with individuals with ASD and their differentiation with respect to their peer group. Examples are difficulties in social interactions and communication or the risk of being the object of jokes and attacks due to their rigidity (repetitive and restricted behaviours and thoughts), lack of assertiveness, possible intense emotional and/or behavioural reactions, their isolation, naiveté, eccentricity, difficulties with mental abilities, and so forth. For example, Cappadocia et al. [32] found direct associations between bullying and some behaviours that can be observed in autism (e.g., stereotypic behaviour, self-injury, communication difficulties, and hypersensitivity), as well as with symptoms of anxiety and hyperactivity, in a sample of 192 parents of children with ASD aged between 5 and 21 years (54% having HFASD).

This becomes worse when individuals with HFASD, perhaps from their possible lack of understanding of the negative intention of the abusive interaction (due to their mental difficulties: repetitive and restricted behaviours and thoughts), reinforce the abuse by responding submissively to the aggressor (e.g., obeying a humiliating order because they want to feel closer to the peer group). Another way they can reinforce the abuse is by not defending themselves against aggressions (e.g., agreeing with a hurtful ironic statement about themselves, due to their difficulties with the indirect uses of language, in turn connected with their repetitive and restricted behaviours and thoughts). In addition, as the study by the Storch team indicated, we can even find a two-way relationship between the signs of anxiety in the individual with ASD facing an episode that generates even more anxiety when it happens, which will reinforce the aggressor as to the possibility of a further attack and so on, successively [79]. These authors found positive relationships between peer abuse and depressive symptoms, loneliness, and anxiety in the 60 participants aged between 11 and 14 years (11 with HFASD) studied.

Another related question is the response that this collective has to such adverse life events. It is not clear, for example, if the individuals with the highest functioning respond more seriously to negative events than the general population does or than individuals with different degrees of intellectual disability do [40] or whether, at least, they do so in a different way [68]. This has traditionally been explained by the fact that this group seems to be more vulnerable than control group individuals to developing affective disorders and depressive symptoms [80], and this vulnerability seems to be correlated with a genetic predisposition [68]. Ghaziuddin and colleagues [40] argue that autistic response to negative events with depressive symptoms is likely to stem from the fact that they are genetically predisposed to depression. However, no systematic studies have been performed to evaluate the degree to which response to life events and onset of depression in this group is mediated by genetic factors.

An issue that often arises involves the domain of repetitive and restricted behaviours. On the basis of answers given by 89 parents of children aged 5 to 17 and diagnosed with ASD, Stratis and Lecavalier [81] found that adolescents with high levels of ritualistic and sameness behaviour tend to show more severe symptoms of depression. They furthermore concluded that high levels of restricted interests are associated with less severe symptoms of depression and argued that restricted interests may be a protective factor against depression in individuals with ASD.

*(g) Biological Factors and Comorbidity.* As we already pointed out regarding the risk factor discussed in the first section* (Histories of First-Degree Relatives and Environmental Context)*, the question arises as to whether there are any biological factors, that is, neurotransmitters, or character structure that contribute to the pathophysiology of depression in ASD. Indeed, a correlation mechanism between ASD and depression has been identified [26, 45]. Such findings may be in accordance with evidence from a recent genetic study examining risk loci that demonstrates shared effects on ASD, attention deficit/hyperactivity disorder, schizophrenia, bipolar disorder, and major depressive disorder [26].

Equally well-known is the fact that serotonin (monoamine neurotransmitter) is involved in conditions like depression and ASD. Several findings show that changes in serotonin levels can influence the brain through various mechanisms and support the hypothesis that serotonin plays a major role in the development of mood disorders and ASD [82]. The question is whether it is a cause or rather an effect of these phenomena.

The involvement of genetic factors is evident from the results of a twin study, while many gene variants that seem to affect brain development and synaptic functions have been reported in association with ASD [38, 83].

From the reviewed literature we may conclude that even though there is a promising research line on underlying biological mechanisms that may possibly be shared by both ASD and mood disorders, the scientific evidence regarding this question is not yet strong enough.

With regard to comorbidity [7], several studies deal with the cooccurrence of ASD and a number of psychiatric disorders (e.g., clinical youth aged 7–17 that had been diagnosed with mood or anxiety disorders, etc.). Pine et al. [84] observed that 57% of youth patients with bipolar disorder, 38% with major depressive disorder, and 25% with anxiety disorder likewise presented ASD-related symptoms. These findings suggest that ASD is closely associated with mood alterations in pediatric patients, although the etiological relevance of this correlation is unclear [85].

### 3.3. Implications of Depression in Individuals with HFASD: Risk of Suicide

There is presently a renewed interest in the importance of studying and assessing suicide. The American Psychiatric Association has developed some guides for preventing and assessing suicide risk. It also recommends, in the 5th edition of the DSM, that the presence or absence of suicide risk should be assessed as a sixth separate axis (“V 02 Suicidal Behaviour Disorder” [1, 86, 87]).

In the general population, suicide ranks among the top causes of death in adolescents and is tending to grow [88]. However, studies usually focus on cases of completed suicide, which leads to an underestimation of the true prevalence. In individuals with ASD, systematic studies focusing on suicidal behaviours are rare and have been carried out with very small samples, sometimes even with just 1 or 2 cases (see [89–91]). We know that the risk of suicide in individuals with ASD is also underestimated. This may stem from the low index of completed suicide in children and preadolescents and from the fact that ASD represents one of the most commonly forgotten diagnoses in adult psychiatry [24, 89]. Furthermore, the pathognomonic features of ASD can mask the symptoms that indicate immediate risk of suicide. Deterioration in communication and social interaction, inappropriate or strange behaviours, cognitive deficits, and negative symptoms can make this difficult to detect when assessing individuals with ASD [92]. Nevertheless, it is essential to evaluate this risk in this collective. One reason for this is the high prevalence of suicidal ideation and attempts. Another (as some studies indicate) is that these individuals are more likely to complete the suicide successfully on their first attempt, especially when they are adults [93] and they perform the act using violent methods (e.g., hanging, shooting or poisoning themselves, or jumping [94]).

Among the few epidemiological studies on suicide and ASD, we can point out the one by Balfe and Tantam [95]. They studied 42 adolescents and adults with HFASD (mean age, 26.21 years) and found that 15% had attempted suicide. The team of Storch reported that approximately 11% of the 102 youngsters aged between 7 and 16 years with HFASD studied showed suicidal ideation and behaviours associated with depression and posttraumatic stress disorder [25]. The team of Raja also provided similar data when they studied 26 adult patients with ASD and psychotic comorbidity, reporting 7.7% of suicides (2 cases), 3.8% suicide attempts (1 case), 3.8% self-injury (1 case), and 30.8% (8 cases) with suicidal ideation [92]. In reference to this last behaviour (suicidal ideation), Shtayermman [96] found it in 50% (5 cases) of adolescents and young adults with autism; and Wing [12] indicated that 18.75% (3 cases) of a sample with AS showed suicidal ideation or attempts. Such thoughts are significantly present to a greater degree in children with autism than in the NT [97].

In other studies on suicide in this collective, the working procedure is the reverse; ASD is diagnosed starting with populations admitted to hospital for suicide attempt. For example, in the study by the team for Høg with 126 children, 97% (123 cases) were diagnosed with at least one psychiatric diagnosis and, among them, 7 children presented psychosis and ASD [98]. In their retrospective study, Mikami's team found that, of 94 adolescents aged less than 20 years, 12.8% (12 cases) presented ASD [99]. In a similar study, Kato et al. [93] studied 587 patients aged over 18 and found that 7.3% (43 cases) presented ASD. In all these studies, the prevalence of individuals with ASD was much higher than that found in the general population.

In young NTs, poor interpersonal problem-solving skills, impulsiveness and aggressive behaviour have been linked to a greater risk of suicidal behaviour [99]. There is also evidence that depression constitutes a risk factor for suicide attempts among adolescent NTs [27, 100]. Higher-functioning individuals with ASD can attempt suicide or complete a suicide, especially when experiencing depressive symptoms. In fact, as the team for Mayes concluded [73], depression and, to a lesser degree, behavioural problems and school bullying are among the psychological problems most highly predictive of suicidal ideation or suicide attempts. Specifically, this team found that suicidal ideation and attempts were 28 times more frequent in children of up to 16 years old with autism (14%, *n* = 791; 537 with HFASD) than in NT children (0.5%, *n* = 186) but 3 times less frequent than in children that were depressed (43%, *n* = 35). Around 50% of mothers of children with autism reported depression and 77% of the children with suicidal ideation or attempts were considered depressed by their mothers. The researchers concluded that certain demographic variables (age of 10 years or older, male, black, or Hispanic ethnic group, low standing of father's professional activity, and history of school bullying) constituted factors of risk with suicidal ideation and attempts in autism. They also concluded that behavioural problems (disobedience, rebellion, and aggressivity), impulsiveness, depression, and mood dysregulation (explosive, irritable, and with tantrums; [73]) were likewise factors of risk in the same way.

This team found that suicidal ideation or suicide attempts were 3 times more frequent in children that suffered school bullying than in children that did not [73]. The team for Mikami [99] also identified conflicts in personal relationships, such as being a victim of bullying, as the main events precipitating suicide attempts in their sample of adolescents with ASD. Nine out of 12 psychiatric patients hospitalised after a suicide attempt had this problem. The researchers interpreted that these patients, due to their rigid thought patterns and lack of imagination, had difficulties in developing relationships with peers and that the feelings of isolation due to the lack of social support seemed to be the most common psychosocial factors predisposing to suicide attempts in adolescents with ASD [99]. In the NT population, studies such as that by the team for Cui [101] concluded that victimisation from bullying was a significant predictor of subjective perception of suicidal ideation and attempts in their sample of 8,778 Chinese adolescents.

Traditionally, suicide attempts in HFASD have been described as more frequent in adolescence or early adulthood [12, 94]. However, such attempts have also been registered in adults (at the age of 23 years: [102]) (at that of 44 years: [103]) and children of more than 10 years [73]. Cases of completed suicide are rare in younger children [104]. This greater frequency of suicidal acts in adolescence has been related to possible intimidation and feelings of inadequacy in facing all the social demands produced in puberty [94]. It could be assumed that these stressful situations have to last over time, given that some studies indicate that their sample was less prone to attempt suicide based on incidents that had happened in the previous 24 h [93]. This finding led them to suggest that occasional stressful factors had less explicative strength in the group with ASD compared to the control group (adult patients that had also attempted suicide).

Consequently, the most accepted factors of risk for suicide in individuals with ASD [24, 25] include different variables (see Table 2).

### 3.4. Detecting Depression in Individuals with HFASD: Assessment Difficulties and Proposals

Based on our review of the studies to identify and assess depression in individuals with ASD, we can conclude that the symptoms described in the literature include the behavioural indicators most used for the diagnosis of depression in the general population. However, the presentation of depression varies considerably in this collective. Apart from the most generalised symptoms of persistent sadness and loss of interest in activities, depressed autistic individuals may present certain unique features, manifesting depression in a nontypical manner.

For that reason, symptoms from the DSM-5 for major depressive disorder [1, Box 1] are included, with special emphasis on those most related to this collective.

In addition to these DSM-included symptoms, studies on depression in individuals with HFASD include the following as the most representative: (1) increase in behaviours that indicate poor adaptation, including irritability, self-aggression, and heteroaggression [21, 105–108]; (2) recurrent thoughts about death (not just fear of death), suicidal ideation, and suicide plans or attempts [107]; (3) low self-esteem and/or reduced abilities of self-care, hygiene, general appearance, or work performance [107]; (4) decrease or lack of interest in their special interests and rituals and so forth or change in the frequency of their obsessive-compulsive, repetitive, and stereotypic behaviours [107].

With respect to the last symptom, we know that anxiety and obsessive-compulsive spectrum disorders (OCSD) are highly comorbid in this population (see [109]). In fact, growing evidence supports a phenomenological overlap between OCSD and ASD [110]. However, there is no consensus as to whether the presence of depression increases or decreases these symptoms in individuals with ASD. The some authors argue that individuals with ASD and depression present a significant increase in symptoms related to anxiety and OCD, such as stereotypic behaviour, obsessions and/or compulsions and self-destructive behaviours [35, 107]. Authors such as Perry et al. [111] indicate that depression not only increases this nucleus of symptoms, but also can exacerbate the pathology in the rest of core autistic characteristics (decreased communication, language, and social interaction). On the opposite side of the debate, traditional studies such as Gillberg [112] or, more currently, the Mazzone team [68] postulate that the onset of depression in individuals with HFASD is generally (although not always) associated with a decrease in repetitive and obsessive behaviours. When this remission occurs, there is a risk that it might be considered an improvement of the nuclear symptomatology instead of a concurrent depressive state, masking depression even more in this group.

The disorder of ASD often involves the presentation of symptoms that are hard to differentiate from depressive symptoms; these can overlap and go undetected among the core syndrome symptomatology (communicative and social isolation and mental rigidity) or associated symptoms (e.g., sleep and eating disorders [106, 107]). However, in cases with ASD in which these behaviours appear, they remain in a chronic form, not episodic as in depression. In contrast, the presence of fluctuations in behaviour and a stable negative variation in mood unrelated to environmental changes helps to discern that the change is more likely to be the onset of depression and not from a variation in the environment that affects the individual's “desire for sameness” [11], which would translate into greater agitation, uneasiness, or episodic stress.

This process becomes even more complicated, given that diagnosing depression depends primarily on verbal skills and communication of symptoms. In this collective, individuals find it hard to express feelings such as sadness, to carry out reciprocal conversations, speak about themselves, use abilities of the theory of mind, and feel empathy for their own feelings or those of others; for that reason, the individuals affected may not be able to interpret or communicate many of the key characteristics of depression [68, 113]. These difficulties in reporting on their moods could be considered similar to those found in individuals with intellectual restriction, but in autism, in which alexithymia is often found [53], these features are present independently of cognitive level [107]. In fact, there is evidence that young people with HFASD tend to report fewer depressive symptoms than those they really present [79] and they do so in terms related to deterioration of cognition, language, behaviour, or activity more than in terms related to their mood [114]. Along these lines, when parents' assessments, self-reports, and self-assessment of emotional symptoms in children were compared, the children with ASD reported significantly lower levels of depression than those indicated by the parents of 54 students with HFASD [115], with children aged from 7 to 13 years [116] or children from 7 to 13 years [48]. The results of the Mazefsky team with 38 children aged from 10 to 17 years with HFASD led them to conclude that it was necessary to be very cautious in interpreting the scores of individuals with HFASD when evaluating a possible diagnostic comorbidity using self-reports [117]. Specifically, when evaluating depression rates using the short form of the Children's Depression Inventory (Children's Depression Inventory-Short; CDI-S; [118]), they observed a high rate of false negatives, with lower sensitivity and specificity than in the typical population.

The difficulty existing in diagnosing this type of comorbidity is well documented, but it is necessary to consider this possible concurrence to establish appropriate treatment for children and adolescents with HFASD in a situation of risk. The approach to depression and the risk of suicide in youngsters with HFASD includes techniques of prevention and modification of risk factors, along with therapeutic intervention once the risk is detected. There are many interventions available to improve the quality of life of individuals with ASD [119, 120], including social skills training, behavioural therapy, and cognitive-behavioural therapy, which reduce the affective problems in children with HFASD [121, 122]. Treatment should also include interventions aimed at alleviating the coexisting problems that can add to depression and/or suicidal ideation and attempts, such as mockery, behavioural problems, impulsivity, mood dysregulation, and stress [73]. Some studies suggest that serotonin reuptake inhibitors improve mood in cases of ASD [123, 124]. However, there are no clear conclusions on the impact of these therapies in depressive symptoms or their efficacy in children with ASD having lower cognitive functioning [17].

## 4. Discussion and Conclusions

In this study we have reviewed the reports published on children and adolescents with ASD in general and on those with HFASD in particular. We now turn to presenting and discussing the possible implications of the study performed, bearing in mind the study objectives: (1) presenting the* depression rates* in children and adolescents with HFASD; (2) analysing the possible risk factors and explicative hypotheses for diagnostic and/or symptomatological comorbidity of HFASD and depression collected mainly from the literature, and establishing a possible working hypothesis supported by the profiles of potential vulnerability and contributing factors; (3) evaluating the implications of depression in HFASD (the* risk of suicide*); and (4) analysing the difficulties for* assessment* of depression (and risk of suicide) in children and adolescents with HFASD and proposing alternatives.

(1) As for* prevalence *rates, all the studies ratify that depression seems to be common in cases of ASD. However, until now, it has been difficult to establish a generic prevalence rate (see Table 2). No study has shown conclusive results based on a representative sample of individuals with ASD in general or with HFASD in particular. That is why there is a need for large-scale longitudinal population studies, which not only would make it possible to establish incidence, but would also help to identify the type, prognosis, duration, and treatment of depression in ASD. Likewise, such studies would help to ascertain its incidence based on variables such as cognitive functioning or autistic symptomatology, gender, and age.

With respect to this, it is important to highlight some* methodological* problems and limitations found in the studies on depression in autism. Comparing results between the different studies turns out to be difficult, because the sample characteristics vary considerably. For example, they differ in age and gender of the participants, diagnostic criteria and methods of sampling, as well as in symptom assessment in both depression (diagnostic or symptomatological) and in ASD itself (cognitive and symptomatological levels of individuals with ASD). This makes it hard to be able to establish comparisons and generalisations from these data.

(2) As for* explicative hypotheses and risk factors*, we have been able to establish the variables that are related the most according to the literature. Although there is not sufficient empirical evidence for any of these factors, the results can be of help in prevention and early detection.

Our working hypothesis was confirmed, although the results of the studies carried out to date are scant. It seems that the individuals with ASD and a higher cognitive level are more vulnerable to depression and that depression can put the person with ASD at risk of suicide. This higher level of cognitive functioning is, in turn, related to the individual's greater awareness of his/her own limitations, their impact, and implications, and greater capacity for introspection and skills to self-report the symptoms [17].

(3) Concerning* depressive symptomatology*, in the results of the review performed, which gave rise to the proposal for indicators that facilitate detecting depression in this collective (see Table 1), we can see that individuals with ASD that are depressed can show a wide range of symptoms ranging from irritability and sadness to aggressive outbursts and suicide [40]. In the few published studies on suicide in cases of ASD, depression and social relationships with a history of abuse are reported to be psychosocial factors that are especially relevant for early suicide detection. Given that suffering peer bullying is very frequent in this population, watchfulness and intervention in the face of possible cases in children and adolescents with HFASD are important and they are essential when a repeat history of this type of abuse is detected. Insofar as biological factors, the research is less conclusive. For example, gender and ethnic differences in ASD cases are a bit inconsistent in the samples of adolescents, while suicidal ideation is found more often in children, with no effect by gender on suicide attempts. Both ideation and attempts are significantly more frequent among minors of black and Hispanic ethnicity [73].

The high index of depression in this collective emphasises the need to detect suicidal tendencies. We also know that a previous attempt should not minimise the concern about the risk of suicide, because many individuals commit suicide on their first or second attempt [125]. Assessment of the risk of suicide is difficult in this population and can lead to error, suicidal attempts are very frequent in this collective [92], and individuals with ASD do not always give information about themselves or confide in others when experiencing depressed moods [107]. Consequently, it is imperative to maintain a high level of suspicion during clinical evaluation or treatment. This is especially true when there is a history of a recent change in the level of functioning, particularly around the time of puberty.

(4) With respect to the* difficulties in evaluating the indicators for detection of depression in individuals with HFASD*, it is hard to define indicators and criteria for a differential diagnosis of mood disorders (see Box 1), due to the overlap between the symptoms of HFASD and depression [107]. Another difficulty is the characteristics themselves of individuals with ASD, such as alterations in verbal and nonverbal communication, which can affect how the person expresses her/his depressive symptoms, especially when using self-assessment. For that reason, it is essential to establish adequately both the criteria on which the* assessment techniques* employed are based [126–128] and the* informants* (in most studies, the individuals with HFASD themselves or their relatives).

As for the first aspect (assessment techniques), there are currently no scales designed specifically for evaluating this collective or psychiatric comorbidity in general and depression specifically (neither by third-party information or by self-reports). That is why instruments, questionnaires, lists of verification/control, and scales, designed for use in the general population or in the population with learning disabilities have been used in the majority of the studies, or the researchers have turned to the diagnostic criteria in the DSM-IV-TR [2]. Three of the most widely used scales for diagnosing and evaluating the severity of depression and which can constitute a reference for adapting them for this collective are Hamilton's depression scale (Depression Rating Scale [129]), the short form of the Children's Depression Inventory (Children's Depression Inventory-Short; CDI-S; [118]), and the Beck Depression Inventory [130].

All these instruments possess various limitations, as they have not been adapted specifically to take into consideration the neuropsychological characteristics of individuals with ASD with respect to their difficulties in identifying, processing, understanding, and communicating their own inner states, feelings, and emotions, as well as their problems with reciprocal communication and possible alexithymia. The instruments include questions in which the individuals are asked to subjectively evaluate their mood and how they feel. These abilities can be compromised in this collective, with subsequent decreases in the sensitivity of the clinical evaluation. In some sections in the assessment can also focus on alterations related to appetite, sleep, interest in activities, and psychomotor delay; these can be masked by some of the symptoms associated with autism and others that are important in individuals with ASD, such as maladaptive behaviours, can be neglected in evaluation [107].

Consequently, in the absence of specific psychometric instruments to evaluate comorbid psychopathological symptoms in ASD, an alternative strategy could be gathering information of different configurations, including both directed observation and multiple evaluations with rating scales [36] by the individuals themselves as well as by the rest of the informants. The team for Stewart [107] proposes using the diagnostic criteria for psychiatric disorders for adults having intellectual disability/mental retardation (DC-LD: Diagnostic Criteria-Learning Disabilities; [131]), although recognising that these criteria should also be modified for use in individuals with ASD. Furthermore, given that inventories and rating scales are generally inflexible, they do not permit exploring answers and run the risk of poor interpretation by informants with ASD. For these reasons, future evaluations should attempt to complement the results from these tools with structured clinical interviews that make it possible to explore replies and ask for in-depth clarification, bearing in mind the specific difficulties of this collective (e.g., literal interpretation of questions, intersubjective difficulties with the interviewer, etc.).

Concerning the second aspect (the informants), using multiple sources is recommended [14]. It would be important to contrast the relatives' information and that of the affected individuals themselves with that of the teachers and/or professionals working with the individuals with HFASD [132]. This could help to establish whether the parents' perceptions are consistent with the professionals', who have greater longitudinal experience with human development. It would also permit data triangulation, given that parents usually report significantly greater emotional symptoms than the children with HFASD themselves [48, 115, 116]. All of this should bear in mind that, in NT individuals, parents and teachers are unable to estimate the symptoms of sadness and anxiety appropriately and cannot identify internalising disorders [133, 134].

As for* possible paths of future research*, which would complement, refute, or confirm the studies carried out to date, we have seen that depression is a disorder that affects the functioning and quality of life of the person with HFASD. It has important specific consequences, among which is the risk of suicide, and can make the pathognomonic symptomatology of ASD worse. Prevention is, therefore, crucial. It can be carried out on three levels: (1) promoting research that examines or reaches conclusions on risk factors in greater detail, which would make it possible to focus preventative efforts on the most vulnerable populations with ASD; (2) increasing the number of studies that improve the capability of early detection once the depressive episode starts or symptoms appear; and (3) investigating the effect of intervention focused on the advancement and improvement of social relationships from the first years, promoting self-esteem, and on the conditions for cognitive and emotional development of the children. Specifically, it would be reasonable and effective to evaluate how interventions on attributions, self-concept, and social information processing influence depression in individuals with ASD [135]. Future studies in this field would help us to confirm whether this type of interventions is the most appropriate for both preventing the appearance and improving the evolution of depression. It would also allow the empirical establishment of the relative weight of variables such as risk factors in the onset and evolution of depression and risk of suicide in ASD. This would be made possible by verifying if depressive symptoms and suicide decrease when these individuals improve their social and communication difficulties.

To perform these new studies it will be necessary to establish new instruments for the specific assessment of affective disorders in ASD, tools that facilitate improvements in prevention, identification, and treatment. These should not only prove their validity, but also show their specificity for differential diagnosis and assessment of depression in individuals with ASD, resolving the difficulty that standardised administration generates in this population [107] . To accomplish this and in agreement with the needs detected and analysed in our work, large-scale population studies that are descriptive, epidemiological, and etiological and by intervention should be carried out. Such studies would make more in-depth research possible on incidence and risk groups, symptomatological expression, differential diagnosis, duration, prognosis and treatment of depression, and prevention of suicide, in the individuals with ASD.

## Figures and Tables

**Box 1:**
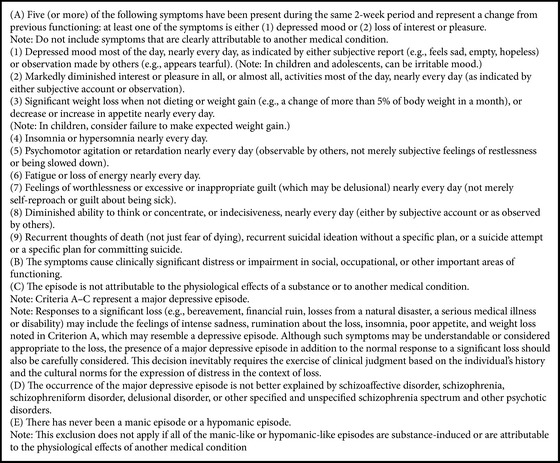
Diagnostic criteria for major depressive disorder in the DSM-5 [1, pages 160–165].

**Table 1:** Results on prevalence rates, risk factors, and explicative hypotheses for depression in ASD.

Study	Sample: *n* (mean), years, age range	Diagnosis	Assessment of depression	Results on prevalence rates of depression	Risk factors and explicative hypotheses
Gillberg and Billstedt (2000) [7]	*n* = 35 (age: DK) Range: children and adolescents with autism	ASD	Review of the literature	33% had an additional psychiatric disorder, with depression being the most common diagnosis	Biological factors: comorbid conditions may be markers for underlying pathophysiology

Whitehouse et al. (2009) [10]	*n* = 35 AS (age: 14.2) Range: 12–17.6 CG: *n* = 35 NT (age: 14.4) Range: 13.2–16.10	HFASD	CES-DC (*Centre for Epidemiological Studies Depression Scale*)	33% self-reported significantly higher levels of depressive symptoms than the NT population	Social support: quality of social relationships

Kanner (1943) [11]	*n* = 11 (age: 5.4) Range: 2.4–11	ASD	DK	One showed tendency towards depression.	Social support: quality of social relationships

Wing (1981) [12]	*n* = 34 (age: DK) Range: 5–35	HFASD	DK	The most common psychiatric diagnosis was depression (10 subjects: approximately)	Age; cognitive level, capacity for introspection, awareness of deficits (insight), and alexithymia; life events and effects brought about by character came from the domain of repetitive and restricted behaviours

Ghaziuddin et al. (1998) [13]	*n* = 35 (age: 15) Range: 8–51	HFASD	K–SADS–E (*Kiddie-Schedule for Affective Disorders and Schizophrenia-Epidemiological Version*)	65% had an additional psychiatric disorder, with depression being the most common (37%)	Histories of first-degree relatives and environmental context

Kanne et al. (2009) [14]	*n* = 177 (age: 7.3) Range: 3–18 CG: their siblings (*n* = 148)	Autism	CBCL (*Child Behaviour Checklist*) C-TRF (*Caregiver/Teacher Report Form*)	26% presented depression	Cognitive level; histories of first-degree relatives environmental context

Barnhill and Myles (2001) [15]	*n* = 33 AS (age: 15) Range: 12–17	HFASD	CDI (*Children's Depression Inventory*)	54% showed depressive symptoms	Cognitive level, capacity for introspection, awareness of deficits (insight), and alexithymia

Leyfer et al. (2006) [16]	*n* = 109 (age: 9.2) Range: 5–17	Autism	ACI-PL (*Autism Comorbidity Interview-Present and Lifetime Version*): this is a modification on the Kiddie Schedule for Affective Disorders and Schizophrenia	13% major depression	Biological factors: comorbidity

Mayes et al. (2011) [17]	*n* = 627 (age: 6.6) Range: 1–17	ASD (64.4% HFASD)	PBS (Pediatric Behaviour Scale)	The maternal descriptions indicated depression in 72% of the HFASD cases	Gender; age; cognitive level, capacity for introspection, awareness of deficits (insight), and alexithymia; social support: quality of social relationships

Mayes et al. (2011) [18]	*n* = 233 (age: 8.3) Range: 6–16	HFASD (IQ > 79)	PBS	54% of the mothers reported depression in their children	Age; cognitive level, capacity for introspection, awareness of deficits (insight), and alexithymia

Green et al. (2000) [19]	*n* = 20 AS (age: 13.75) Range: 11–19 CG: *n* = 20 (age: 14.47) Range: 11–19	HFASD	ICD-10 (*Tenth Revision of the International Classification of Disease*)	Higher levels of depression than in the CG. Although only 5% satisfied criteria for major depression, 40% showed chronic unhappiness and 55%, irritability	Biological factors: comorbidity

Hurtig et al. (2009) [20]	*n* = 43 AS or HFA (age: 13) Range: 11–17 GC: *n* = 217 (age: 13.5)	HFASD	YSR (*Youth Self-Report*) CBCL (*Child Behaviour Checklist*) TRF (*Teacher Report Form*)	33% self-reported significantly higher levels of depressive symptoms than the NT population	Gender

Kim et al. (2000) [21]	*n* = 59 (19 AS; 40 HFASD) (age: 12) Range: 9–14 CG: *n* = 1751 Range: 9–14	HFASD	OCHS-R (*Ontario Child Health Study-Revised*)	17% significant clinical symptomatology of depression	Biological factors: comorbidity

Meyer et al. (2006) [22]	*n* = 31 AS age: 10.1 Range: 8–14 CG: *n* = 33 NT	HFASD	BASC-SRP (*Behaviour Assessment System for Children-Self Report of Personality*) BASC-PRS (*Behaviour Assessment System for Children-Parent Report Scale*)	Self-reported symptoms of depression higher than in CG	Cognitive level, capacity for introspection, awareness of deficits (insight), and alexithymia

Brereton et al. (2006) [23]	*n* = 381/367 ASD (age: 7.4) Range: 3.8–24 GC: *n* = 581 intellectual disability without ASD	ASD	DBC-P (*Developmental Behaviour Checklist*)	Parents offered significantly higher scores for behaviour problems, anxiety, depression, and irritability compared with normality, as well as higher degrees of anxiety, behaviour problems, depression, and attention-deficit/hyperactivity disorder than in CG	Gender; age

ASD: Autism Spectrum Disorder; AS: Asperger Syndrome; CG: control group; HFASD: high functioning autism spectrum disorder (IQ > 70); NT: neurotypical.

DK: it indicates that the symptom/sign was not discussed in the paper, not that the authors were unable to assess it.

**Table 2:** Overview of factors of risk for suicide in Autism Spectrum Disorder (ASD). Most vulnerable population with ASD (modified from [24, 25]).

	Factors and variables	Studies
Biological	Genetic and biological factors (adolescence, gender, and ethnicity)	[26–28]
	History of relatives (psychiatric disorder or suicide)	[27, 28]
	Substance abuse	[28]
	Comorbidity	Anxiety [29] Depression [25, 27, 28, 30]

Psychological	Impulsiveness	[31]
	Higher cognitive, social development, and communication levels	[24]
	Life events and effects brought by characters came from domain of repetitive and restricted behaviours and thoughts: frequent abuse and school bullying	[27, 32–34]

Social	Poorer social support networks: social isolation	[35]

## Abbreviations

AS: Asperger Syndrome
ASD: Autism Spectrum Disorder
HFASD: High functioning autism spectrum disorder
NT: Neurotypical
OCSD: Obsessive-compulsive spectrum disorders.

## References

[1] American Psychiatric Association (2013). *DSM-5: Diagnostic and Statistical Manual of Mental Disorders*.

[2] American Psychiatric Association (2002). *Diagnostic and Statistical Manual of Mental Disorders*.

[3] Belinchón M., Olivar J. S. (2003). Autistic Spectrum Disorders in (relative) hight-functioning children and adolescents: functional differentiation through multivariate analysis. *Acción Psicológica*.

[4] Howlin P. (2003). Outcome in high-functioning adults with autism with and without early language delays: implications for the differentiation between autism and asperger syndrome. *Journal of Autism and Developmental Disorders*.

[5] Mayes S. D., Calhoun S. L., Murray M. J., Pearl A., Black A., Tierney C. D. (2014). Final DSM-5 under-identifies mild Autism Spectrum Disorder: agreement between the DSM-5, CARS, CASD, and clinical diagnoses. *Research in Autism Spectrum Disorders*.

[6] Matson J. L., Williams L. W. (2014). The making of a field: the development of comorbid psychopathology research for persons with intellectual disabilities and autism. *Research in Developmental Disabilities*.

[7] Gillberg C., Billstedt E. (2000). Autism and Asperger syndrome: coexistence with other clinical disorders. *Acta Psychiatrica Scandinavica*.

[8] Simonoff E., Pickles A., Charman T., Chandler S., Loucas T., Baird G. (2008). Psychiatric disorders in children with autism spectrum disorders: prevalence, comorbidity, and associated factors in a population-derived sample. *Journal of the American Academy of Child and Adolescent Psychiatry*.

[9] Matson J. L., Nebel-Schwalm M. S. (2007). Comorbid psychopathology with autism spectrum disorder in children: an overview. *Research in Developmental Disabilities*.

[10] Whitehouse A. J. O., Durkin K., Jaquet E., Ziatas K. (2009). Friendship, loneliness and depression in adolescents with Asperger's Syndrome. *Journal of Adolescence*.

[11] Kanner L. (1943). Autistic disturbances of affective contact. *Nervous Child*.

[12] Wing L. (1981). Asperger's syndrome: A clinical account. *Psychological Medicine*.

[13] Ghaziuddin M., Weidmer-Mikhail E., Ghaziuddin N. (1998). Comorbidity of Asperger syndrome: a preliminary report. *Journal of Intellectual Disability Research*.

[14] Kanne S. M., Abbacchi A. M., Constantino J. N. (2009). Multi-informant ratings of psychiatric symptom severity in children with autism spectrum disorders: the importance of environmental context. *Journal of Autism and Developmental Disorders*.

[15] Barnhill G. P., Myles B. S. (2001). Attributional style and depression in adolescents with Asperger syndrome. *Journal of Positive Behavior Interventions*.

[16] Leyfer O. T., Folstein S. E., Bacalman S. (2006). Comorbid psychiatric disorders in children with autism: interview development and rates of disorders. *Journal of Autism and Developmental Disorders*.

[17] Mayes S. D., Calhoun S. L., Murray M. J., Zahid J. (2011). Variables associated with anxiety and depression in children with autism. *Journal of Developmental and Physical Disabilities*.

[18] Mayes S. D., Calhoun S. L., Murray M. J., Ahuja M., Smith L. A. (2011). Anxiety, depression, and irritability in children with autism relative to other neuropsychiatric disorders and typical development. *Research in Autism Spectrum Disorders*.

[19] Green J., Gilchrist A., Burton D., Cox A. (2000). Social and psychiatric functioning in adolescents with Asperger syndrome compared with conduct disorder. *Journal of Autism and Developmental Disorders*.

[20] Hurtig T., Kuusikko S., Mattila M. L. (2009). Multi-informant reports of psychiatric symptoms among high functioning adolescents with Asperger syndrome or autism. *Autism*.

[21] Kim J. A., Szatmari P., Bryson S. E., Streiner D. L., Wilson F. J. (2000). The prevalence of anxiety and mood problems among children with autism and Asperger syndrome. *Autism*.

[22] Meyer J. A., Mundy P. C., van Hecke A. V., Durocher J. S. (2006). Social attribution processes and comorbid psychiatric symptoms in children with Asperger syndrome. *Autism*.

[23] Brereton A. V., Tonge B. J., Einfeld S. L. (2006). Psychopathology in children and adolescents with autism compared to young people with intellectual disability. *Journal of Autism and Developmental Disorders*.

[24] Hannon G., Taylor E. P. (2013). Suicidal behaviour in adolescents and young adults with ASD: findings from a systematic review. *Clinical Psychology Review*.

[25] Storch E. A., Sulkowski M. L., Nadeau J. (2013). The phenomenology and clinical correlates of suicidal thoughts and behaviors in youth with autism spectrum disorders. *Journal of Autism and Developmental Disorders*.

[26] Smoller J. W., Craddock N., Kendler K. (2013). Identification of risk loci with shared effects on five major psychiatric disorders: a genome-wide analysis. *The Lancet*.

[27] Steele M. M., Doey T. (2007). Suicidal behaviour in children and adolescents. Part 1: etiology and risk factors. *Canadian Journal of Psychiatry*.

[28] Hawton K., James A. (2005). Suicide and deliberate self harm in young people. *British Medical Journal*.

[29] van Steensel F. J. A., Bögels S. M., Perrin S. (2011). Anxiety disorders in children and adolescents with autistic spectrum disorders: a meta-analysis. *Clinical Child and Family Psychology Review*.

[30] Strang J. F., Kenworthy L., Daniolos P. (2012). Depression and anxiety symptoms in children and adolescents with autism spectrum disorders without intellectual disability. *Research in Autism Spectrum Disorders*.

[31] Mosconi M. W., Cody-Hazlett H., Poe M. D., Gerig G., Gimpel-Smith R., Piven J. (2009). Longitudinal study of amygdala volume and joint attention in 2- to 4-year-old children with autism. *Archives of General Psychiatry*.

[32] Cappadocia M. C., Weiss J. A., Pepler D. (2012). Bullying experiences among children and youth with autism spectrum disorders. *Journal of Autism and Developmental Disorders*.

[33] Van Roekel E., Scholte R. H. J., Didden R. (2010). Bullying among adolescents with autism spectrum disorders: prevalence and perception. *Journal of Autism and Developmental Disorders*.

[34] Sofronoff K., Dark E., Stone V. (2011). Social vulnerability and bullying in children with Asperger syndrome. *Autism*.

[35] Bauminger N., Kasari C. (2000). Loneliness and friendship in high-functioning children with autism. *Child Development*.

[36] White S. W., Roberson-Nay R. (2009). Anxiety, social deficits, and loneliness in youth with autism spectrum disorders. *Journal of Autism and Developmental Disorders*.

[37] Cappe E., Bobet R., Gattegno M. P., Fernier A., Adrien J. L. (2009). Effet d'un programme spécialisé pour enfants autistes sur la qualité de vie de leurs parents. *Revue Québécoise de Psychologie*.

[38] Ijichi S., Ijichi N., Ijichi Y. (2013). Quantitative nature of social vulnerability and autism: an important paradigm shift in the DSM-5 for autism spectrum disorder. *ISRN Neurology*.

[39] Wing L. (1996). Autistic spectrum disorders. *British Medical Journal*.

[40] Ghaziuddin M., Ghaziuddin N., Greden J. (2002). Depression in persons with autism: implications for research and clinical care. *Journal of Autism and Developmental Disorders*.

[41] Sullivan P. F., Neale M. C., Kendler K. S. (2000). Genetic epidemiology of major depression: review and meta-analysis. *The American Journal of Psychiatry*.

[42] Ingersoll B., Hambrick D. Z. (2011). The relationship between the broader autism phenotype, child severity, and stress and depression in parents of children with autism spectrum disorders. *Research in Autism Spectrum Disorders*.

[43] Piven J., Palmer P. (1999). Psychiatric disorder and the broad autism phenotype: evidence from a family study of multiple-incidence autism families. *The American Journal of Psychiatry*.

[44] Micali N., Chakrabarti S., Fombonne E. (2004). The broad autism phenotype: findings from an epidemiological survey. *Autism*.

[45] Cook E. H., Charak D. A., Arida J., Spohn J. A., Roizen N. J. M., Leventhal B. L. (1994). Depressive and obsessive-compulsive symptoms in hyperserotonemic parents of children with autistic disorder. *Psychiatry Research*.

[46] Rezendes D. L., Scarpa A. (2011). Associations between parental anxiety/depression and child behavior problems related to autism spectrum disorders: the roles of parenting stress and parenting self-efficacy. *Autism Research and Treatment*.

[47] Sukhodolsky D. G., Scahill L., Gadow K. D. (2008). Parent-rated anxiety symptoms in children with pervasive developmental disorders: frequency and association with core autism symptoms and cognitive functioning. *Journal of Abnormal Child Psychology*.

[48] Vickerstaff S., Heriot S., Wong M., Lopes A., Dossetor D. (2007). Intellectual ability, self-perceived social competence, and depressive symptomatology in children with high-functioning autistic spectrum disorders. *Journal of Autism and Developmental Disorders*.

[49] Mazurek M. O., Kanne S. M. (2010). Friendship and internalizing symptoms among children and adolescents with ASD. *Journal of Autism and Developmental Disorders*.

[50] Tantam D. (2000). Psychological disorder in adolescents and adults with Asperger syndrome. *Autism*.

[51] Gotham K., Bishop S. L., Brunwasser S., Lord C. (2014). Rumination and perceived impairment associated with depressive symptoms in a verbal adolescent-adult ASD sample. *Autism Research*.

[52] Hedley D., Young R. (2006). Social comparison processes and depressive symptoms in children and adolescents with Asperger syndrome. *Autism*.

[53] Berthoz S., Hill E. L. (2005). The validity of using self-reports to assess emotion regulation abilities in adults with autism spectrum disorder. *European Psychiatry*.

[54] Zackheim L. (2007). Alexithymia: the expanding realm of research. *Journal of Psychosomatic Research*.

[55] Fitzgerald M., Bellgrove M. A. (2006). The overlap between alexithymia and asperger's syndrome. *Journal of Autism and Developmental Disorders*.

[56] Del Barrio M. V. (2007). *El niño deprimido: Causas, evaluación y tratamiento*.

[57] Hartup W. W., Stevens N. (1999). Friendships and adaptation across the life span. *Current Directions in Psychological Science*.

[58] Butzer B., Konstantareas M. M. (2003). Depression, temperament and their relationship to other characteristics in children with Asperger syndrome. *Journal on Developmental Disabilities*.

[59] Sofronoff K., Attwood T., Hinton S. (2005). A randomised controlled trial of a CBT intervention for anxiety in children with Asperger syndrome. *Journal of Child Psychology and Psychiatry*.

[60] Ozonoff S., Dawson G., McPartland J. (2002). *A Parent’s Guide to Asperger Syndrome and High-Functioning Autism*.

[61] Caldwell M. S., Rudolph K. D., Troop-Gordon W., Kim D.-Y. (2004). Reciprocal influences among relational self-views, social disengagement, and peer stress during early adolescence. *Child Development*.

[62] Troop-Gordon W., Ladd G. W. (2005). Trajectories of peer victimization and perceptions of the self and schoolmates: precursors to internalizing and externalizing problems. *Child Development*.

[63] Prinstein M. J., Aikins J. W. (2004). Cognitive moderators of the longitudinal association between peer rejection and adolescent depressive symptoms. *Journal of Abnormal Child Psychology*.

[64] Linsey E. W. (2002). Preschool children's friendships and peer acceptance: links to social competence. *Child Study Journal*.

[65] Bauminger N., Shulman C., Agam G. (2003). Peer interaction and loneliness in high-functioning children with autism. *Journal of Autism and Developmental Disorders*.

[66] Bauminger N., Shulman C., Agam G. (2004). The link between perceptions of self and of social relationships in high-functioning children with autism. *Journal of Developmental and Physical Disabilities*.

[67] Lasgaard M., Nielsen A., Eriksen M. E., Goossens L. (2010). Loneliness and social support in adolescent boys with autism spectrum disorders. *Journal of Autism and Developmental Disorders*.

[68] Mazzone L., Ruta L., Reale L. (2012). Psychiatric comorbidities in Asperger syndrome and high functioning autism: diagnostic challenges. *Annals of General Psychiatry*.

[69] Ghaziuddin M. (2005). A family history study of asperger syndrome. *Journal of Autism and Developmental Disorders*.

[70] Ghaziuddin M., Alessi N., Greden J. F. (1995). Life events and depression in children with pervasive developmental disorders. *Journal of Autism and Developmental Disorders*.

[71] Hawker D. S. J., Boulton M. J. (2000). Twenty years' research on peer victimization and psychosocial maladjustment: a meta-analytic review of cross-sectional studies. *Journal of Child Psychology and Psychiatry and Allied Disciplines*.

[72] Carter S. (2009). Bullying of students with asperger syndrome. *Issues in Comprehensive Pediatric Nursing*.

[73] Mayes S. D., Gorman A. A., Hillwig-Garcia J., Syed E. (2013). Suicide ideation and attempts in children with autism. *Research in Autism Spectrum Disorders*.

[74] Wainscot J. J., Naylor P., Sutcliffe P., Tantam D., Williams J. V. (2008). Relationships with peers and use of the school environment of mainstream secondary school pupils with asperger syndrome (high-functioning autism): a case-control study. *International Journal of Psychology and Psychological Therapy*.

[75] Little L. (2001). Peer victimization of children with asperger spectrum disorders. *Journal of the American Academy of Child and Adolescent Psychiatry*.

[76] Little L. (2002). Middle-class mothers' perceptions of peer and sibling victimization among children with Asperger's syndrome and nonverbal learning disorders. *Issues in Comprehensive Pediatric Nursing*.

[77] Twyman K. A., Saylor C. F., Saia D., MacIas M. M., Taylor L. A., Spratt E. (2010). Bullying and ostracism experiences in children with special health care needs. *Journal of Developmental and Behavioral Pediatrics*.

[78] Morton J. F., Campbell J. M. (2008). Information source affects peers' initial attitudes toward autism. *Research in Developmental Disabilities*.

[79] Storch E. A., Larson M. J., Ehrenreich-May J. (2012). Peer victimization in youth with autism spectrum disorders and co-occurring anxiety: relations with psychopathology and loneliness. *Journal of Developmental and Physical Disabilities*.

[80] Attwood T. (2006). *The Complete Guide to Asperger's Syndrome*.

[81] Stratis E. A., Lecavalier L. (2013). Restricted and repetitive behaviors and psychiatric symptoms in youth with autism spectrum disorders. *Research in Autism Spectrum Disorders*.

[82] Kepser L. J., Homberg J. R. (2015). The neurodevelopmental effects of serotonin: a behavioural perspective. *Behavioural Brain Research*.

[83] Betancur C. (2011). Etiological heterogeneity in autism spectrum disorders: more than 100 genetic and genomic disorders and still counting. *Brain Research*.

[84] Pine D. S., Guyer A. E., Goldwin M., Towbin K. A., Leibenluft E. (2008). Autism spectrum disorder scale scores in pediatric mood and anxiety disorders. *Journal of the American Academy of Child & Adolescent Psychiatry*.

[85] Matsuo J., Kamio Y., Takahashi H. (2015). Autistic-like traits in adult patients with mood disorders and schizophrenia. *PLOS ONE*.

[86] Oquendo M. A., Currier D., Posner K. (2009). Reconceptualizing psychiatric nosology: the case of suicidal behaviour. *Revista de Psiquiatria y Salud Mental*.

[87] García-Nieto R., Uribe I. P., Palao D. (2012). Protocolo breve de evaluación del suicidio: fiabilidad interexaminadores [Brief suicide questionnaire. Inter-rater reliability]. *Revista de Psiquiatría y Salud Mental*.

[88] Hawton K., Saunders K. E. A., O'Connor R. C. (2012). Self-harm and suicide in adolescents. *The Lancet*.

[89] Fitzgerald M. (2007). Suicide and Asperger's syndrome. *Crisis*.

[90] Barry-Walsh J. B., Mullen P. E. (2004). Forensic aspects of Asperger's syndrome. *Journal of Forensic Psychiatry and Psychology*.

[91] Frazier J. A., Doyle R., Chiu S., Coyle J. T. (2002). Treating a child with Asperger's disorder and comorbid bipolar disorder. *American Journal of Psychiatry*.

[92] Raja M., Azzoni A., Frustaci A. (2011). Autism spectrum disorders and suicidality. *Clinical Practice and Epidemiology in Mental Health*.

[93] Kato K., Mikami K., Akama F. (2013). Clinical features of suicide attempts in adults with autism spectrum disorders. *General Hospital Psychiatry*.

[94] Gillberg C. (2002). *A Guide to Asperger Syndrome*.

[95] Balfe M., Tantam D. A. (2010). A descriptive social and health profile of a community sample of adults and adolescents with Asperger syndrome. *BMC Research Notes*.

[96] Shtayermman O. (2007). Peer victimization in adolescents and young adults diagnosed with Asperger's Syndrome: a link to depressive symptomatology, anxiety symptomatology and suicidal ideation. *Issues in Comprehensive Pediatric Nursing*.

[97] Gadow K. D., Guttmann-Steinmetz S., Rieffe C., DeVincent C. J. (2012). Depression symptoms in boys with autism spectrum disorder and comparison samples. *Journal of Autism and Developmental Disorders*.

[98] Høg V., Isager T., Skovgaard A. M. (2002). Suicidal behaviour in children—a descriptive study. *Ugeskrift for Laeger*.

[99] Mikami K., Inomata S., Hayakawa N. (2009). Frequency and clinical features of pervasive developmental disorder in adolescent suicide attempts. *General Hospital Psychiatry*.

[100] Gould M. S., Greenberg T., Velting D. M., Shaffer D. (2003). Youth suicide risk and preventive interventions: a review of the past 10 years. *Journal of the American Academy of Child and Adolescent Psychiatry*.

[101] Cui S., Cheng Y., Xu Z., Chen D., Wang Y. (2011). Peer relationships and suicide ideation and attempts among Chinese adolescents. *Child: Care, Health and Development*.

[102] Mikami K., Ohya A., Akasaka K., Matsumoto H. (2006). Attempted suicide of youth with Asperger's disorder. *Seishinigaku Zasshi*.

[103] Spencer L., Lyketsos C. G., Samstad E., Dokey A., Rostov D., Chisolm M. S. (2011). A suicidal adult in crisis: an unexpected diagnosis of autism spectrum disorder. *The American Journal of Psychiatry*.

[104] Windfuhr K., While D., Hunt I. (2008). Suicide in juveniles and adolescents in the United Kingdom. *Journal of Child Psychology and Psychiatry and Allied Disciplines*.

[105] Long K., Wood H., Holmes N. (2000). Presentation, assessment and treatment of depression in a young woman with learning disability and autism. *British Journal of Learning Disabilities*.

[106] Mayes S. D., Calhoun S. L. (1999). Symptoms of autism in young children and correspondence with the DSM. *Infants and Young Children*.

[107] Stewart M. E., Barnard L., Pearson J., Hasan R., O'Brien G. (2006). Presentation of depression in autism and Asperger syndrome: a review. *Autism*.

[108] Turygin N. C., Matson J. L., MacMillan K., Konst M. (2013). The relationship between challenging behavior and symptoms of depression in intellectually disabled adults with and without Autism spectrum disorders. *Journal of Developmental and Physical Disabilities*.

[109] De-la-Iglesia M., Olivar J.-S. (2012). Revision of studies and researches related with diagnostic comorbidity of autism spectrum disorder-high functioning and anxiety disorders. *Anales de Psicología, Norteamérica*.

[110] Chasson G. S., Timpano K. R., Greenberg J. L., Shaw A., Singer T., Wilhelm S. (2011). Shared social competence impairment: another link between the obsessive-compulsive and autism spectrums?. *Clinical Psychology Review*.

[111] Perry D. W., Marston G. M., Hinder S. A. J., Munden A. C., Roy A. (2001). The phenomenology of depressive illness in people with a learning disability and autism. *Autism*.

[112] Gillberg C. (1985). Asperger's syndrome and recurrent psychosis: a case study. *Journal of Autism and Developmental Disorders*.

[113] Sanders J. L. (2009). Qualitative or quantitative differences between Asperger's disorder and autism? Historical considerations. *Journal of Autism and Developmental Disorders*.

[114] Lainhart J. E., Folstein S. E. (1994). Affective disorders in people with autism: a review of published cases. *Journal of Autism and Developmental Disorders*.

[115] Nicpon M. F., Doobay A. F., Assouline S. G. (2010). Parent, teacher, and self perceptions of psychosocial functioning in intellectually gifted children and adolescents with autism spectrum disorder. *Journal of Autism and Developmental Disorders*.

[116] Lopata C., Thomeer M. L., Volker M. A. (2010). RCT of a manualized social treatment for high-functioning autism spectrum disorders. *Journal of Autism and Developmental Disorders*.

[117] Mazefsky C. A., Kao J., Oswald D. P. (2011). Preliminary evidence suggesting caution in the use of psychiatric self-report measures with adolescents with high-functioning autism spectrum disorders. *Research in Autism Spectrum Disorders*.

[118] Kovacs M. (1992). *Children's Depression Inventory CDI Manual*.

[119] Adrien J. L., Gattegno M. P. (2011). *Autisme de l’enfant. Evaluations, interventions et suivis*.

[120] Gattegno M., de Fenoyl C. (2004). L’entraînement aux habiletés sociales chez les personnes atteintes de syndrome d’Asperger. *Journal de Thérapie Comportementale et Cognitive*.

[121] Chalfant A. M., Rapee R., Carroll L. (2007). Treating anxiety disorders in children with high functioning autism spectrum disorders: a controlled trial. *Journal of Autism and Developmental Disorders*.

[122] Wood J. J., Drahota A., Sze K., Har K., Chiu A., Langer D. A. (2009). Cognitive behavioral therapy for anxiety in children with autism spectrum disorders: a randomized, controlled trial. *Journal of Child Psychology and Psychiatry and Allied Disciplines*.

[123] Posey D. J., Erickson C. A., Stigler K. A., McDougle C. J. (2006). The use of selective serotonin reuptake inhibitors in autism and related disorders. *Journal of Child and Adolescent Psychopharmacology*.

[124] West L., Brunssen S. H., Waldrop J. (2009). Review of the evidence for treatment of children with autism with selective serotonin reuptake inhibitors. *Journal for Specialists in Pediatric Nursing*.

[125] Janicak P. G., Davis J. M., Preskorn S. H., Ayd F. J., Marder S. R., Pavuluri M. N. (2006). *Principles and Practice of Psychopharmacology*.

[126] Westman Andersson G. W., Miniscalco C., Gillberg C. (2013). Autism in preschoolers: does individual clinician's first visit diagnosis agree with final comprehensive diagnosis?. *The Scientific World Journal*.

[127] Westman Andersson G., Miniscalco C., Johansson U., Gillberg C. (2013). Autism in toddlers: can observation in preschool yield the same information as autism assessment in a specialised clinic?. *The Scientific World Journal*.

[128] Pennington M. L., Cullinan D., Southern L. B. (2014). Defining autism: variability in state education agency definitions of and evaluations for autism spectrum disorders. *Autism Research and Treatment*.

[129] Hamilton M. (1960). A rating scale for depression. *Journal of Neurology, Neurosurgery, and Psychiatry*.

[130] Beck A. T., Ward C. H., Mendelson M., Mock J., Erbaugh J. (1961). An inventory for measuring depression. *Archives of General Psychiatry*.

[131] Bailey N. M., Andrews T. M. (2003). Diagnostic criteria for psychiatric disorders for use with adults with learning disabilities/mental retardation (DC-LD) and the diagnosis of anxiety disorders: a review. *Journal of Intellectual Disability Research*.

[132] Krieger A. E., Saïas T., Adrien J. L. (2013). Promouvoir le partenariat parents-professionnels dans la prise en charge des enfants atteints d'autisme. *L'Encéphale*.

[133] Cova F., Maganto C., Melipillán R. (2005). Género, adversidad familiar y síntomas emocionales en preadolescentes. *Psykhe*.

[134] López C., Alcántara M., Fernández V., Castro M., López J. (2010). Características y prevalencia de los problemas de ansiedad, depresión y quejas somáticas en una muestra clínica infantil de 8 a 12 años, mediante el CBCL (Child Behaviour Checklist) [Characteristics and prevalence of anxiety disorders, depression and somatic complaints in a 8 to 12 years old child clinical sample using the CBCL (Child Behaviour Checklist)]. *Anales de Psicología, Norteamérica*.

[135] Paula-Pérez I., Martos-Pérez J. (2009). Síndrome de Asperger y autismo de alto funcionamiento: Comorbilidad con trastornos de ansiedad y del estado de ánimo. *Revista de Neurología*.

